# Poly[bis­(*N*,*N*-dimethyl­formamide)tris­(μ_4_-*trans*-stilbene-4,4′-dicarboxyl­ato)­tricadmium(II)]: a two-dimensional network with an unusual 3^6^ topology

**DOI:** 10.1107/S1600536808016267

**Published:** 2008-06-07

**Authors:** Dong-Heon Lee, Gyungse Park

**Affiliations:** a Department of Chemistry, Chonbuk National University, Jeonju, Chonbuk 561-756, Republic of Korea; b Department of Chemistry, Kunsan National University, Kusan, Chonbuk 573-701, Republic of Korea

## Abstract

In the title compound, [Cd_3_(C_16_H_10_O_4_)_3_(C_3_H_7_NO)_2_]_*n*_ or [Cd_3_(SDA)_3_(DMF)_2_]_*n*_ (H_2_SDA is *trans*-stilbene-4,4′-dicarboxylic acid and DMF is dimethyl­formamide), the linear dicarboxylate ligand forms a two-dimensionally layered metal–organic network with the relatively uncommon 3^6^ topology. The structure reveals trinuclear secondary building units and has an octa­hedral geometry at a central metal ion (occupying a 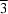 symmetry site) and tetra­hedral geometries at two surrounding symmetrically equivalent metal ions lying on a threefold axis. The six-connected planar trinuclear Cd^II^ centers, Cd_3_(O_2_C*R*)_6_, play a role as potential nodes in generation of the relatively uncommon 3^6^ topology. The coordinated DMF unit is disordered around the threefold axis.

## Related literature

For related literature, see: Chi *et al.* (2006 ▶); Dincâ & Long (2005 ▶); Dybtsev *et al.* (2004 ▶); Eddaoudi *et al.* (2002 ▶); Edgar *et al.* (2001 ▶); Hawxwell *et al.* (2006 ▶); Hill *et al.* (2005 ▶); Luan *et al.* (2006 ▶); Park *et al.* (2006 ▶); Rosi *et al.* (2003 ▶); Saalfrank *et al.* (2001 ▶); Seo *et al.* (2000 ▶); Wang *et al.* (2006 ▶); Williams *et al.* (2005 ▶).
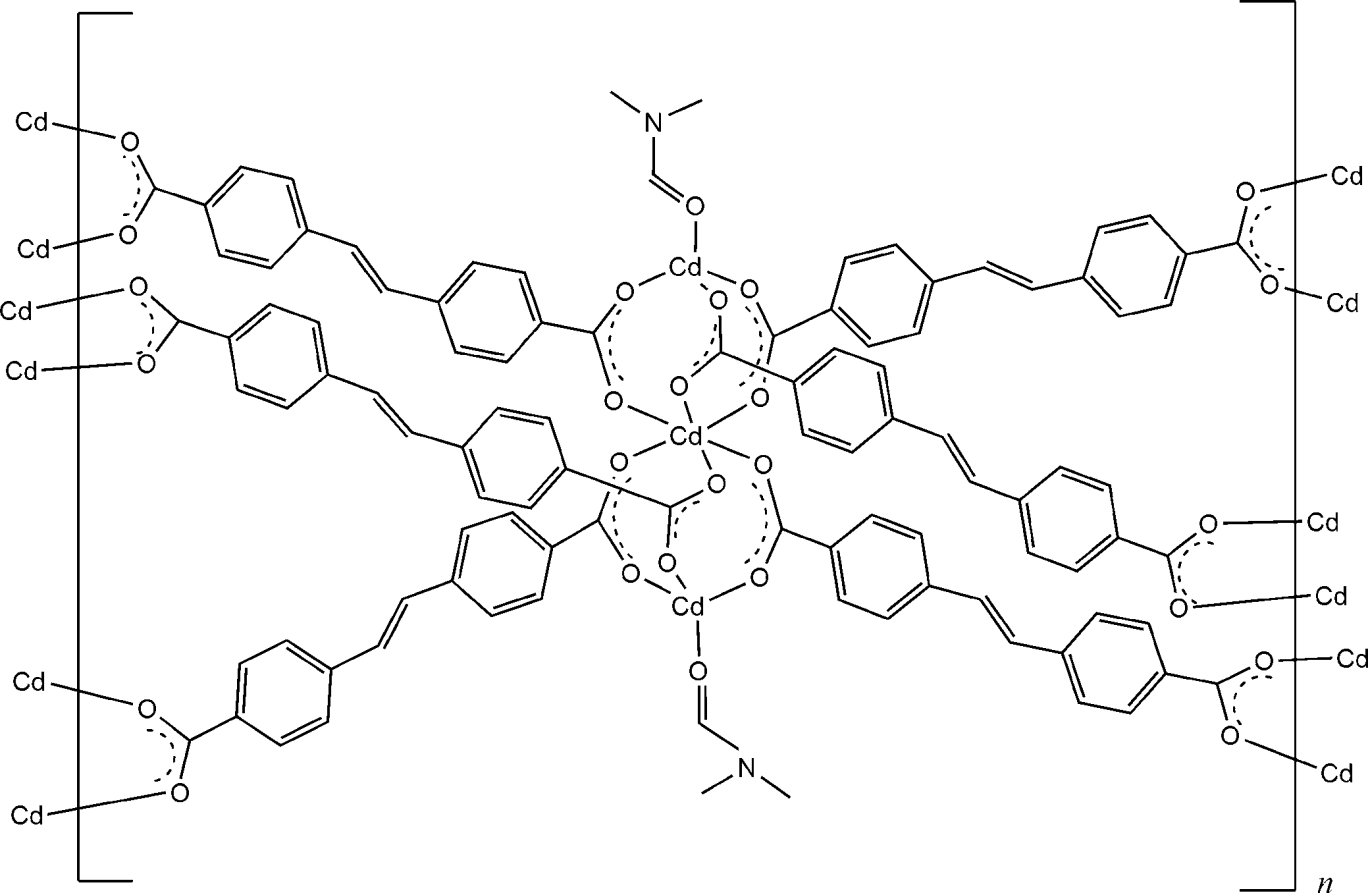


## Experimental

### 

#### Crystal data

[Cd_3_(C_16_H_10_O_4_)_3_(C_3_H_7_NO)_2_]
*M* *_r_* = 1282.11
Trigonal, 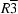

*a* = 16.4881 (5) Å
*c* = 16.7919 (10) Å
*V* = 3953.4 (3) Å^3^
*Z* = 3
Mo *K*α radiation
μ = 1.27 mm^−1^
*T* = 223 (2) K
0.30 × 0.30 × 0.30 mm

#### Data collection

Bruker SMART CCD diffractometer
Absorption correction: multi-scan (*SADABS*; Sheldrick, 1996 ▶) *T*_min_ = 0.69, *T*_max_ = 0.69
6604 measured reflections
2105 independent reflections
1782 reflections with *I* > 2σ(*I*)
*R*_int_ = 0.104

#### Refinement

*R*[*F* ^2^ > 2σ(*F* ^2^)] = 0.057
*wR*(*F* ^2^) = 0.181
*S* = 1.18
2105 reflections
136 parameters
92 restraints
H-atom parameters constrained
Δρ_max_ = 1.70 e Å^−3^
Δρ_min_ = −1.53 e Å^−3^

### 

Data collection: *SMART* (Bruker, 1997 ▶); cell refinement: *SAINT* (Bruker, 1997 ▶); data reduction: *SAINT*; program(s) used to solve structure: *SHELXS97* (Sheldrick, 2008 ▶); program(s) used to refine structure: *SHELXL97* (Sheldrick, 2008 ▶); molecular graphics: *SHELXTL* (Sheldrick, 2008 ▶); software used to prepare material for publication: *SHELXTL*.

## Supplementary Material

Crystal structure: contains datablocks I, global. DOI: 10.1107/S1600536808016267/bg2189sup1.cif

Structure factors: contains datablocks I. DOI: 10.1107/S1600536808016267/bg2189Isup2.hkl

Additional supplementary materials:  crystallographic information; 3D view; checkCIF report

## Figures and Tables

**Fig. 1.**
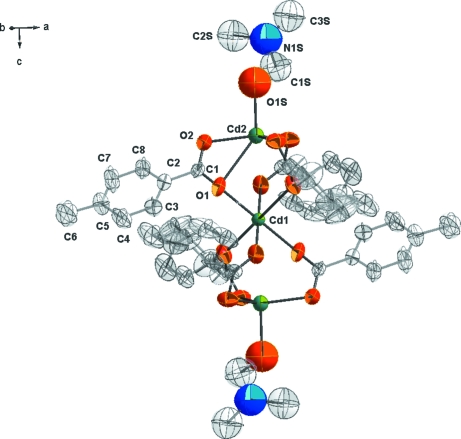
The trinuclear Cd3(O2CR)6 SBU cluster for 1 showing the bridging SDA ligands and the coordinated DMF molecule. The remainder of the SDA is removed and only one of the threefold disordered DMF molecule is shown for clarity. Cd atoms are shown in green, O atoms in red, N atoms in blue and C atoms in grey.

**Fig. 2.**
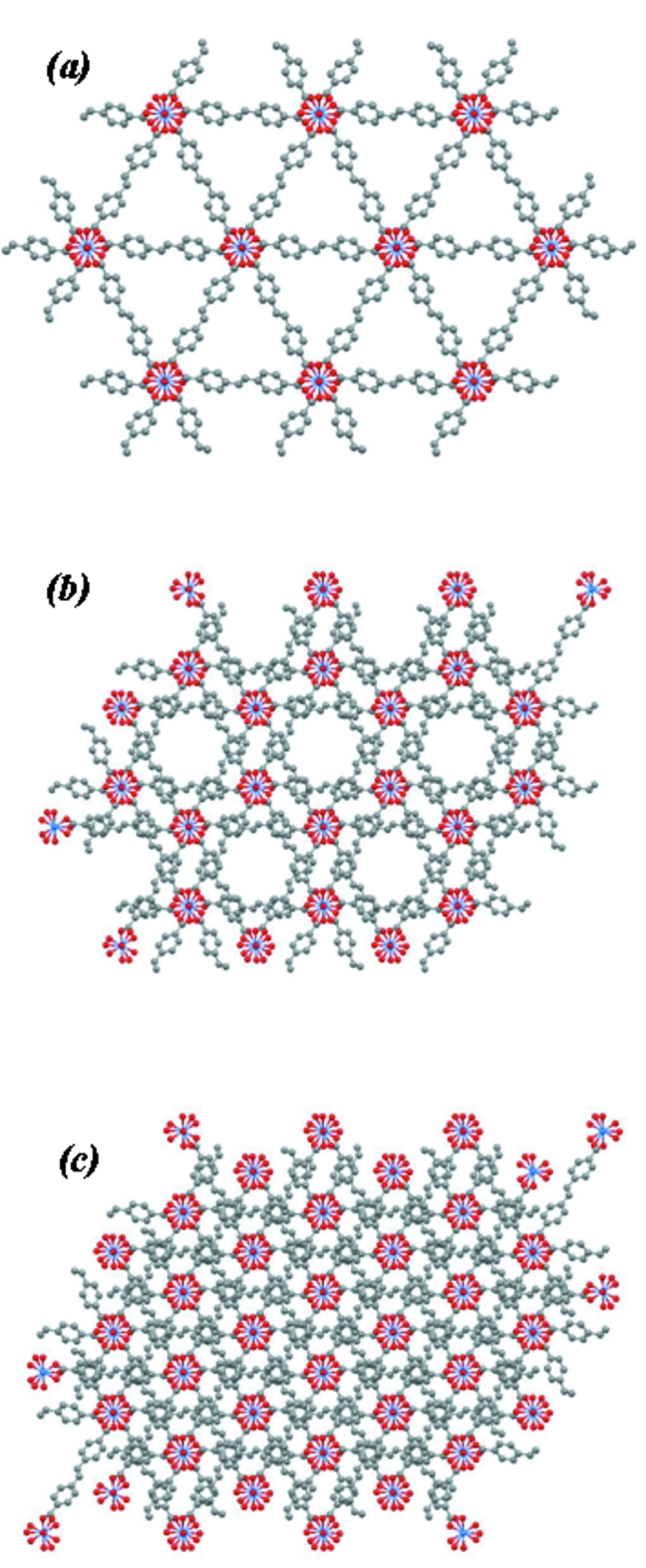
[001] view of the structure showing the 36 topology. (a) A single 2D layer . (b) Two overimposed close-packed layers, A and B. (c) Cubic close-packed layers, in ABC pattern.

## supplementary crystallographic information

### Comment

The study of one, two or three dimensional metal-organic frameworks (MOFs) has attracted much attention in the past decade due to their various intriguing framework topologies but also for their potential applications in gas storage (Rosi *et al.*, 2003), separation (Dybtsev *et al.*, 2004) and catalysis (Seo *et al.*, 2000) etc. Many factors play important role in the synthesis of MOFs such as the coordination geometry of metal ions (Chi *et al.*,2006), the structure of organic ligands (Wang *et al.*,2006), the solvent system (Eddaoudi *et al.*, 2002),the counteranion (Luan *et al.*, 2006), and the ratio of ligands to metal ions (Saalfrank *et al.*, 2001). The simplest 2D sheets are those which comprise just one kind of regular polygon based upon hexagons, squares and triangles. Since three hexagons, four squares and six triangles meet at a node in a 2D network with angles of 120°, 90° and 60°,respectively, the corresponding Schläfli topology symbols are 6^3^, 4^4^ and 3^6^, respectively (Hill *et al.*, 2005). Although there were many examples of uninodal regularly tiled 2D metal–organic frameworks comprising linked squares or hexagons, however, a few examples comprising linked and tiled triangles have been reported only very recently (Edgar *et al.*, 2001; Williams *et al.*, 2005; Hawxwell *et al.*, 2006; Dincâ & Long, 2005). Herein the formation of a two-dimensional metal-organic framework with an uncommon 3^6^ tessellated topology, [Cd_3_(SDA)_3_(DMF)_2_], (I), constructed from tri-nuclear cadmium SBUs (secondary building units) linked by a novel 4,4'-stilbenedicarboxylate ligand (Park *et al.*, 2006) is reported.

The two-dimensional 3^6^ tessellated network structure of 1 with the atomic numbering scheme is shown in Fig. 1 in which the coordinated DMF molecules are shown in only one of its three disordered components. The crystal structure of 1 is constructed from the tri-nuclear Cd_3_(O_2_CR)_6_ SBUs cluster which contains two crystallographically equivalent four-coordinate terminal metal centers (Cd2) in which the O atom (O1S) of the DMF is axially coordinated and a six-coordinate central metal atom (Cd1). The coordination environment around the central Cd^II^ atom, Cd1, in the trinuclear center is an octahedron with all six positions occupied by one carboxylate oxygen, O1, from each half unit of six SDA ligands (Fig. 1) and that of the two symmetry equivalent neighbouring Cd^II^ atoms, Cd2, is a tetrahedron with three coordination sites occupied by the other carboxylate oxygen, O2, from a half unit of three SDA ligands and the vacant site occupied by an oxygen atom, O1S in the DMF molecule.

### Experimental

A mixture of Cd(NO_3_)_2_.6H_2_O (0.122 g, 3.95 x 10 ^-4^ mol) and H_2_SDA (0.106 g,3.95 x 10 ^-4^ mol) was suspended in DMF (1.3 ml), placed in a sealed-glasstube,and heated at 90°C for 3 days. Upon cooling to room temperature, the pale-yellow crystalline was formed, collected by filtration, washed with DMF,and driedunder a reduced pressure at room temperature for 5 h to give the product (0.178 g, 78%). Anal. Calcd. for [Cd_3_(SDA)_3_(DMF)_2_]: C,50.59; H, 3.75; N, 2.18. Found: C, 50.69; H, 3.72; N, 2.12

### Refinement

All the non-hydrogen atoms were refined anisotropically, and hydrogen atoms were added to their geometrically ideal positions with distances C—H = 0.94 Å (aromatic H), C—H = 0.94 Å (attached to carboxylic C in DMF) and C—H = 0.97 Å (attached to methyl C in DMF). Coordinated DMF is disordered over three sites around the threefold axis. Even if oxygen O1S was refined with a unique position, the large displacement factor attained suggests some kind of unresolved splitting. Similarity restraints in distances and thermal parameters were used in order to attain a reasonable geometry of the (disordered) coordinated DMF.

### Crystal data

**Table:**

[Cd_3_(C_16_H_10_O_4_)_3_(C_3_H_7_N_1_O_1_)_2_]	*Z* = 3
*M**_r_* = 1282.11	*F*_000_ = 1914
Trigonal, *R*3	*D*_x_ = 1.616 Mg m^−^^3^
Hall symbol: -R 3	Mo *K*α radiation λ = 0.71073 Å
*a* = 16.4881 (5) Å	Cell parameters from 6604 reflections
*b* = 16.4881 (5) Å	θ = 1.9–28.4º
*c* = 16.7919 (10) Å	µ = 1.27 mm^−^^1^
α = 90º	*T* = 223 (2) K
β = 90º	Cubic, colourless
γ = 120º	0.30 × 0.30 × 0.30 mm
*V* = 3953.4 (3) Å^3^	

### Data collection

**Table:**

Siemens SMART CCD diffractometer	2105 independent reflections
Radiation source: fine-focus sealed tube	1782 reflections with *I* > 2σ(*I*)
Monochromator: graphite	*R*_int_ = 0.104
*T* = 223(2) K	θ_max_ = 28.4º
ω scans	θ_min_ = 1.9º
Absorption correction: multi-scan(SADABS; Sheldrick, 1996)	*h* = −21→21
*T*_min_ = 0.69, *T*_max_ = 0.69	*k* = −21→18
6604 measured reflections	*l* = −21→22

### Refinement

**Table:**

Refinement on *F*^2^	Secondary atom site location: difference Fourier map
Least-squares matrix: full	Hydrogen site location: inferred from neighbouring sites
*R*[*F*^2^ > 2σ(*F*^2^)] = 0.057	H-atom parameters constrained
*wR*(*F*^2^) = 0.181	*w* = 1/[σ^2^(*F*_o_^2^) + (0.0995*P*)^2^ + 4.8382*P*] where *P* = (*F*_o_^2^ + 2*F*_c_^2^)/3
*S* = 1.18	(Δ/σ)_max_ = 0.001
2105 reflections	Δρ_max_ = 1.70 e Å^−^^3^
136 parameters	Δρ_min_ = −1.53 e Å^−^^3^
92 restraints	Extinction correction: none
Primary atom site location: structure-invariant direct methods	

### Special details

**Table:**

Geometry. All e.s.d.'s (except the e.s.d. in the dihedral angle between two l.s. planes) are estimated using the full covariance matrix. The cell e.s.d.'s are taken into account individually in the estimation of e.s.d.'s in distances, angles and torsion angles; correlations between e.s.d.'s in cell parameters are only used when they are defined by crystal symmetry. An approximate (isotropic) treatment of cell e.s.d.'s is used for estimating e.s.d.'s involving l.s. planes.
Refinement. Refinement of F^2^ against ALL reflections. The weighted R-factor wR and goodness of fit S are based on F^2^, conventional R-factors R are based on F, with F set to zero for negative F^2^. The threshold expression of F^2^ > σ(F^2^) is used only for calculating R-factors(gt) etc. and is not relevant to the choice of reflections for refinement. R-factors based on F^2^ are statistically about twice as large as those based on F, and R- factors based on ALL data will be even larger.

### Fractional atomic coordinates and isotropic or equivalent isotropic displacement parameters (Å^2^)

**Table:**

	*x*	*y*	*z*	*U*_iso_*/*U*_eq_	Occ. (<1)
Cd1	0.0000	1.0000	1.0000	0.0302 (2)	
Cd2	0.0000	1.0000	0.79310 (3)	0.0424 (2)	
O1	0.1245 (2)	1.0679 (2)	0.9162 (2)	0.0521 (7)	
O2	0.1222 (2)	1.1401 (2)	0.8067 (2)	0.0599 (9)	
C1	0.1562 (3)	1.1402 (3)	0.8740 (3)	0.0458 (9)	
C2	0.2391 (3)	1.2283 (3)	0.9015 (3)	0.0579 (12)	
C3	0.2624 (5)	1.3134 (4)	0.8665 (4)	0.0748 (17)	
H3A	0.2259	1.3142	0.8238	0.090*	
C4	0.3368 (6)	1.3965 (5)	0.8918 (5)	0.109 (3)	
H4A	0.3496	1.4531	0.8677	0.131*	
C5	0.3911 (6)	1.3967 (5)	0.9510 (5)	0.112 (3)	
C6	0.4698 (8)	1.4929 (7)	0.9730 (7)	0.138 (4)	
H6	0.4748	1.5449	0.9456	0.166*	
C7	0.3714 (7)	1.3114 (7)	0.9864 (6)	0.133 (4)	
H7A	0.4095	1.3120	1.0283	0.159*	
C8	0.2968 (5)	1.2260 (5)	0.9610 (4)	0.099 (3)	
H8A	0.2859	1.1690	0.9831	0.118*	
O1S	0.0000	1.0000	0.6625 (11)	0.186 (4)	
N1S	0.106 (2)	1.082 (2)	0.5571 (18)	0.188 (5)	0.33
C1S	0.067 (4)	1.086 (4)	0.625 (2)	0.190 (6)	0.33
H1S	0.0825	1.1436	0.6479	0.228*	0.33
C2S	0.136 (3)	1.014 (3)	0.547 (3)	0.188 (5)	0.33
H2S1	0.1426	0.9916	0.5985	0.282*	0.33
H2S2	0.1958	1.0429	0.5194	0.282*	0.33
H2S3	0.0899	0.9616	0.5156	0.282*	0.33
C3S	0.144 (3)	1.160 (3)	0.502 (2)	0.190 (5)	0.33
H3S1	0.0964	1.1771	0.4909	0.284*	0.33
H3S2	0.1615	1.1420	0.4531	0.284*	0.33
H3S3	0.1980	1.2129	0.5257	0.284*	0.33

### Atomic displacement parameters (Å^2^)

**Table:**

	*U*^11^	*U*^22^	*U*^33^	*U*^12^	*U*^13^	*U*^23^
Cd1	0.0307 (3)	0.0307 (3)	0.0291 (4)	0.01536 (14)	0.000	0.000
Cd2	0.0414 (3)	0.0414 (3)	0.0443 (4)	0.02070 (14)	0.000	0.000
O1	0.0407 (15)	0.0402 (15)	0.068 (2)	0.0147 (13)	0.0172 (14)	0.0081 (13)
O2	0.0493 (17)	0.061 (2)	0.0463 (17)	0.0106 (15)	0.0029 (13)	0.0043 (14)
C1	0.0361 (19)	0.044 (2)	0.047 (2)	0.0117 (16)	0.0112 (16)	0.0003 (16)
C2	0.052 (3)	0.048 (2)	0.048 (2)	0.006 (2)	0.0016 (19)	0.0048 (18)
C3	0.062 (3)	0.047 (3)	0.090 (4)	0.008 (3)	−0.004 (3)	0.013 (3)
C4	0.093 (5)	0.045 (3)	0.134 (7)	−0.006 (3)	−0.026 (5)	0.008 (4)
C5	0.108 (6)	0.067 (4)	0.091 (5)	−0.008 (4)	−0.016 (4)	−0.006 (4)
C6	0.137 (8)	0.095 (6)	0.130 (8)	0.018 (5)	−0.037 (6)	0.024 (5)
C7	0.113 (7)	0.112 (7)	0.094 (5)	−0.003 (5)	−0.057 (5)	0.013 (5)
C8	0.090 (4)	0.075 (4)	0.075 (4)	−0.001 (3)	−0.032 (3)	0.025 (3)
O1S	0.192 (4)	0.192 (4)	0.174 (6)	0.096 (2)	0.000	0.000
N1S	0.186 (6)	0.190 (6)	0.181 (6)	0.089 (4)	0.000 (4)	0.000 (4)
C1S	0.192 (8)	0.191 (7)	0.178 (7)	0.088 (6)	−0.002 (5)	−0.006 (5)
C2S	0.186 (6)	0.190 (6)	0.183 (6)	0.090 (4)	−0.001 (4)	0.001 (4)
C3S	0.189 (6)	0.190 (6)	0.184 (6)	0.090 (4)	0.000 (4)	0.001 (4)

### Geometric parameters (Å, °)

**Table:**

Cd1—O1^i^	2.269 (3)	C5—C7	1.408 (13)
Cd1—O1^ii^	2.269 (3)	C5—C6	1.509 (12)
Cd1—O1^iii^	2.269 (3)	C6—C6^vi^	1.279 (19)
Cd1—O1	2.269 (3)	C6—H6	0.9400
Cd1—O1^iv^	2.269 (3)	C7—C8	1.395 (10)
Cd1—O1^v^	2.269 (3)	C7—H7A	0.9400
Cd1—Cd2^v^	3.4742 (5)	C8—H8A	0.9400
Cd1—Cd2	3.4742 (5)	O1S—C1S^iii^	1.43 (4)
Cd2—O2	2.189 (3)	O1S—C1S^i^	1.43 (4)
Cd2—O2^iii^	2.189 (3)	O1S—C1S	1.43 (4)
Cd2—O2^i^	2.189 (3)	N1S—C1S	1.323 (9)
Cd2—O1S	2.193 (19)	N1S—C3S	1.445 (9)
O1—C1	1.255 (5)	N1S—C2S	1.448 (9)
O2—C1	1.262 (6)	C1S—H1S	0.9400
C1—C2	1.484 (6)	C2S—H2S1	0.9700
C2—C3	1.386 (8)	C2S—H2S2	0.9700
C2—C8	1.394 (8)	C2S—H2S3	0.9700
C3—C4	1.373 (9)	C3S—H3S1	0.9700
C3—H3A	0.9400	C3S—H3S2	0.9700
C4—C5	1.336 (12)	C3S—H3S3	0.9700
C4—H4A	0.9400		
			
O1^i^—Cd1—O1^ii^	180.00 (13)	C3—C2—C1	120.9 (5)
O1^i^—Cd1—O1^iii^	85.62 (14)	C8—C2—C1	120.1 (5)
O1^ii^—Cd1—O1^iii^	94.38 (14)	C4—C3—C2	122.4 (7)
O1^i^—Cd1—O1	85.62 (14)	C4—C3—H3A	118.8
O1^ii^—Cd1—O1	94.38 (14)	C2—C3—H3A	118.8
O1^iii^—Cd1—O1	85.62 (14)	C5—C4—C3	119.8 (7)
O1^i^—Cd1—O1^iv^	94.38 (14)	C5—C4—H4A	120.1
O1^ii^—Cd1—O1^iv^	85.62 (14)	C3—C4—H4A	120.1
O1^iii^—Cd1—O1^iv^	180.000 (1)	C4—C5—C7	119.5 (6)
O1—Cd1—O1^iv^	94.38 (14)	C4—C5—C6	114.1 (8)
O1^i^—Cd1—O1^v^	94.38 (14)	C7—C5—C6	126.4 (8)
O1^ii^—Cd1—O1^v^	85.62 (14)	C6^vi^—C6—C5	123.3 (13)
O1^iii^—Cd1—O1^v^	94.38 (14)	C6^vi^—C6—H6	118.4
O1—Cd1—O1^v^	180.000 (1)	C5—C6—H6	118.4
O1^iv^—Cd1—O1^v^	85.62 (14)	C8—C7—C5	121.7 (7)
O1^i^—Cd1—Cd2^v^	128.30 (9)	C8—C7—H7A	119.1
O1^ii^—Cd1—Cd2^v^	51.70 (9)	C5—C7—H7A	119.1
O1^iii^—Cd1—Cd2^v^	128.30 (9)	C7—C8—C2	117.4 (7)
O1—Cd1—Cd2^v^	128.30 (9)	C7—C8—H8A	121.3
O1^iv^—Cd1—Cd2^v^	51.70 (9)	C2—C8—H8A	121.3
O1^v^—Cd1—Cd2^v^	51.70 (9)	C1S^iii^—O1S—C1S^i^	102 (3)
O1^i^—Cd1—Cd2	51.70 (9)	C1S^iii^—O1S—C1S	102 (3)
O1^ii^—Cd1—Cd2	128.30 (9)	C1S^i^—O1S—C1S	102 (3)
O1^iii^—Cd1—Cd2	51.70 (9)	C1S^iii^—O1S—Cd2	116 (2)
O1—Cd1—Cd2	51.70 (9)	C1S^i^—O1S—Cd2	116 (2)
O1^iv^—Cd1—Cd2	128.30 (9)	C1S—O1S—Cd2	116 (2)
O1^v^—Cd1—Cd2	128.30 (9)	C1S—N1S—C3S	120.7 (11)
Cd2^v^—Cd1—Cd2	180.0	C1S—N1S—C2S	120.1 (11)
O2—Cd2—O2^iii^	118.93 (3)	C3S—N1S—C2S	116.8 (10)
O2—Cd2—O2^i^	118.93 (3)	N1S—C1S—O1S	119 (4)
O2^iii^—Cd2—O2^i^	118.93 (3)	N1S—C1S—H1S	120.4
O2—Cd2—O1S	95.98 (9)	O1S—C1S—H1S	120.4
O2^iii^—Cd2—O1S	95.98 (9)	N1S—C2S—H2S1	109.5
O2^i^—Cd2—O1S	95.98 (9)	N1S—C2S—H2S2	109.5
O2—Cd2—Cd1	84.02 (9)	H2S1—C2S—H2S2	109.5
O2^iii^—Cd2—Cd1	84.02 (9)	N1S—C2S—H2S3	109.5
O2^i^—Cd2—Cd1	84.02 (9)	H2S1—C2S—H2S3	109.5
O1S—Cd2—Cd1	180.000 (4)	H2S2—C2S—H2S3	109.5
C1—O1—Cd1	131.5 (3)	N1S—C3S—H3S1	109.5
C1—O2—Cd2	105.6 (3)	N1S—C3S—H3S2	109.5
O1—C1—O2	122.1 (4)	H3S1—C3S—H3S2	109.5
O1—C1—C2	119.8 (4)	N1S—C3S—H3S3	109.5
O2—C1—C2	118.1 (4)	H3S1—C3S—H3S3	109.5
C3—C2—C8	119.0 (5)	H3S2—C3S—H3S3	109.5

Symmetry codes: (i) −*x*+*y*−1, −*x*+1, *z*; (ii) *x*−*y*+1, *x*+1, −*z*+2; (iii) −*y*+1, *x*−*y*+2, *z*; (iv) *y*−1, −*x*+*y*, −*z*+2; (v) −*x*, −*y*+2, −*z*+2; (vi) −*x*+1, −*y*+3, −*z*+2.
